# Thyroid Dysfunction and Risk of Parkinson’s Disease: A Systematic Review and Meta-Analysis

**DOI:** 10.3389/fendo.2022.863281

**Published:** 2022-05-04

**Authors:** Nipith Charoenngam, Thanitsara Rittiphairoj, Ben Ponvilawan, Klaorat Prasongdee

**Affiliations:** ^1^ Department of Medicine, Mount Auburn Hospital, Harvard Medical School, Cambridge, MA, United States; ^2^ Department of Medicine, Faculty of Medicine Siriraj Hospital, Mahidol University, Bangkok, Thailand; ^3^ Harvard T.H. Chan School of Public Health, Boston, MA, United States; ^4^ Department of Pharmacology, Faculty of Medicine Siriraj Hospital, Mahidol University, Bangkok, Thailand; ^5^ Department of Medicine, MetroWest Medical Center, Framingham, MA, United States

**Keywords:** thyroid, hyperthyroidism, hypothyroidism, parkinson’s disease, systematic review, meta-analysis

## Abstract

**Objective:**

Studies have suggested that patients with thyroid dysfunction may have an increased risk of developing Parkinson’s disease (PD). However, the results from existing studies are inconsistent. Therefore, we aimed to investigate the association of hypothyroidism and hyperthyroidism with risk of PD using the method of systematic review and meta-analysis.

**Methods:**

Potentially eligible studies were identified from Medline and EMBASE databases from inception to December 2021 using search strategy that comprised of terms for “Thyroid” and “Parkinson’s Disease”. Eligible cohort study must consist of one cohort of patients with hypothyroidism/hyperthyroidism and another cohort of individuals without hypothyroidism/hyperthyroidism. Then, the study must report effect estimates with 95% confidence intervals (95% CIs) comparing incident PD between the groups. Eligible case-control studies must include cases with PD and controls without PD. Then, the study must explore their history of hypothyroidism/hyperthyroidism. Odds ratio (OR) with 95% CIs of the association between presence of hypothyroidism/hyperthyroidism and PD must be reported. Point estimates with standard errors were retrieved from each study and were combined together using the generic inverse variance method.

**Results:**

A total of 3,147 articles were identified. After two rounds of independent review by three investigators, 3 cohort studies and 6 case-control studies met the eligibility criteria and were included into the meta-analysis. Pooled analysis showed an increased likelihood of PD in both patients with hypothyroidism (pooled OR 1.56; 95%CI, 1.38 – 1.77; with moderate heterogeneity, I^2^ 66.9%) and patients with hyperthyroidism (pooled OR 1.57; 95%CI, 1.40 – 1.77; with insignificant heterogeneity, I^2^ 0.0%). Funnel plots for both meta-analyses were fairly symmetric, which did not indicate presence of publication bias.

**Conclusion:**

This systematic review and meta-analysis found a significant association of both hypothyroidism and hyperthyroidism with an increased risk of PD.

## Introduction

Parkinson’s disease (PD) is one of the most common neurodegenerative disorders that causes motor dysfunction including bradykinesia, tremor, rigidity and postural instability as well as neurocognitive impairment and depression (1). This condition affects approximately 0.3% of the general population and is about 1.5 times more common among men than women (2). The histopathological characteristic of PD is degeneration of mesencephalic dopaminergic neurons in the basal ganglia (3). The pathogenesis of the disease is still unclarified; however, it is believed to involve mitochondrial dysfunction and oxidative stress of the neurons (4).

Thyroid hormone plays a crucial role in not only regulating cellular metabolism and multiple organ systems, but also controlling neurodevelopment as well as modulating neurotransmission (5–7). It is well-known that hypothyroidism in early life can cause neurocognitive deficit and that thyroid dysfunction thyroid dysfunction in adult can manifest as neuropsychiatric symptoms such as memory problem and depression in hypothyroidism and irritability, insomnia, anxiety and psychosis in hyperthyroidism (8–10). In addition, evidence from clinical and epidemiologic studies suggests an inverted U-shaped association between thyroid function and cognitive function. In other words, both individuals with hypothyroidism and hyperthyroidism may have an increased risk of cognitive decline and dementia (10, 11).

It is however still unclear whether the presence of thyroid dysfunction can increase the risk of any specific neurodegenerative disorder (12). In fact, a number of observational studies have reported the association between thyroid dysfunction and PD, yielding mixed results (13–21). Using systematic review and meta-analysis technique, we aimed to investigate whether patients with thyroid dysfunction (i.e., hypothyroidism and hyperthyroidism) had an increased risk of PD by identifying all available cohort and case-control studies and summarizing their results together.

## Method

### Search Strategy

Three investigators (N.C., B.P., K.P.) independently searched records indexed in Medline and Embase from inception to December 2021. Search terms were obtained from terms related to “Hypothyroidism”, “Hyperthyroidism” and “Parkinson’s disease”. The detailed search strategy is shown in the **Supplemental Material 1**. No language limitation was applied. To ensure the comprehensiveness of study identification, the literature review was also performed in Google Scholar and bibliography of the eligible studies that were initially identified from EMBASE and MEDLINE. This study was performed in concordance with the Preferred Reporting Items for Systematic Reviews and Meta-Analyses statement, as shown in the **Supplemental Material 2**.

### Inclusion Criteria

Eligible study must be either cohort or case-control study. Eligible cohort study must consist of one cohort of patients with hypothyroidism/hyperthyroidism and another cohort of individuals without hypothyroidism/hyperthyroidism. Then, the study must report effect estimates (e.g., relative risk, incidence rate ratio, hazard risk ratio or standardized incidence ratio) with 95% confidence intervals (95% CIs) of incident PD between individuals with hypothyroidism/hyperthyroidism versus comparators without hypothyroidism/hyperthyroidism. Eligible case-control studies must consist of cases with PD and controls without PD. Then, the study must explore their history of hypothyroidism/hyperthyroidism. Odds ratio (OR) with 95% CIs of the association between presence of hypothyroidism/hyperthyroidism and PD or percentage of participants with hypothyroidism/hyperthyroidism in each group must be reported.

Three investigators (N.C., B.P., K.P) independently reviewed the eligibility of the retrieved articles. Different opinion was resolved by discussion with the senior investigators (N.C., T.R.). Two investigators evaluated the quality of each study (N.C. and T.R.) using the Newcastle-Ottawa quality assessment scale for cohort study and case-control study (22).

### Data Extraction

A standardized collection form was used for data extraction of the following information: last name of the first author, country of the study, study design, publication year, main findings, number of participants, recruitment of participants, diagnosis of hypothyroidism/hyperthyroidism, diagnosis of PD, follow-up duration (for cohort studies), average age of participants, percentage of female participants, comorbidities of participants and variables adjusted in multivariate analysis.

### Statistical Analysis

We performed two separate meta-analyses including 1.) the meta-analysis of the association between hypothyroidism and risk of PD and 2.) the meta-analysis of the association between hyperthyroidism and risk of PD. Effect estimates with standard errors were extracted from each eligible study. Extracted effect estimates were combined together using the generic inverse variance method as described by DerSimonian and Laird (23). Random-effect model was used given that the eligible studies had different study protocols and background populations. Of note, for each eligible study that reported multiple effect estimates from different analysis models, one with most robust adjustment for confounders was selected with the aim to minimize the confounding effects. For those cohort studies that did not report adjusted estimates, number of subjects with outcome in each group would be extracted to calculate odds ratios, which would be combined with those from other case-control studies. For those cohort studies that reported effect estimates with adjustments for confounders (i.e., adjusted standardized incidence ratio or hazard ratio), those estimates would be selected and included into the pooled analysis with the assumption that the provided close estimation of relative likelihood of outcome. 

The Cochran’s Q test was used to assess statistical heterogeneity. This statistical analysis was further complimented by the I^2^ statistic which determines the proportion of the total variation across studies that is secondary to heterogeneity rather than coincidence. A value of I^2^ of 0 – 25% represents insignificant heterogeneity, 26–50% low heterogeneity, 51–75% moderate heterogeneity and >75% high heterogeneity (24). If enough studies qualified for the meta-analysis, visualization of funnel plot would be used for investigating the presence of publication bias. All data analyses were performed using the StataMP15.

## Results

### Search Results

**Figure 1:**
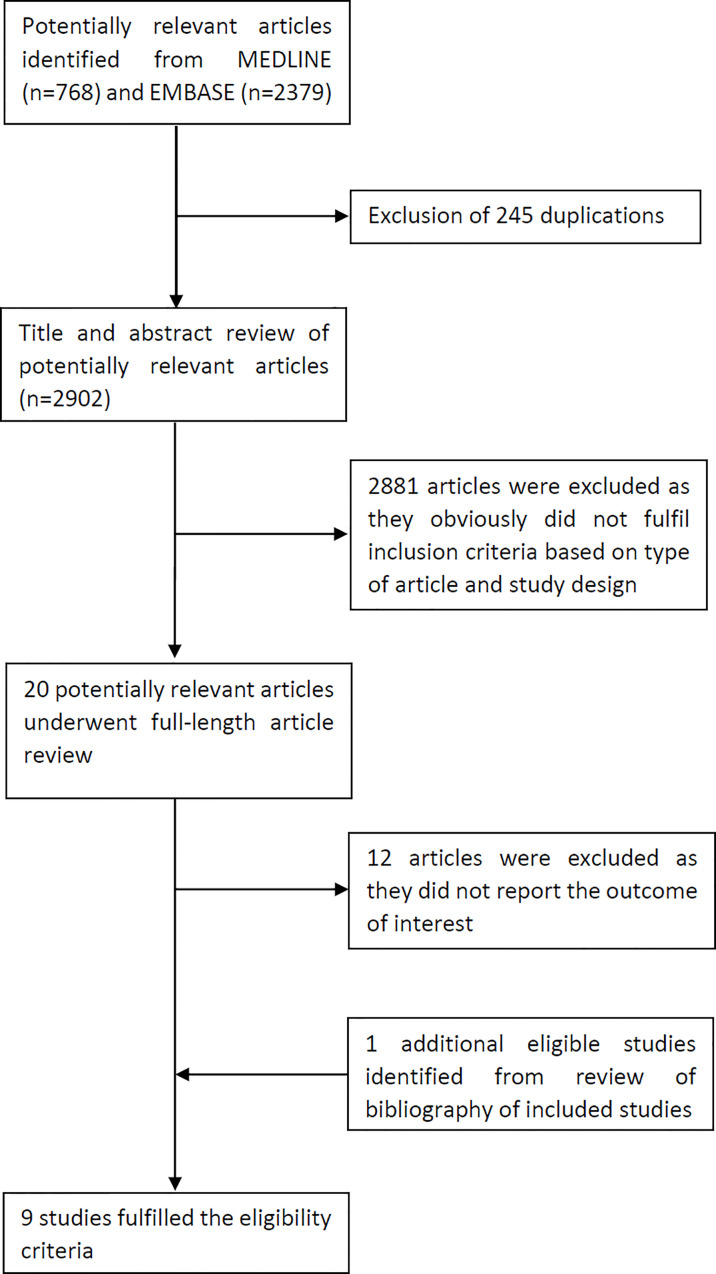
Study identification and literature review process.

A total of 3,147 records were retrieved from EMBASE and Medline databases in which 245 duplicated records were removed, leaving 2,902 articles for review of title and abstract. A total of 2,881 records were further excluded provided that they obviously did not meet the eligibility criteria based on study design and type of article, leaving 20 articles for full-text review. A total of 12 records were excluded at this stage since they did not report the outcome of interest, leaving 8 records eligible for the meta-analysis (13–19, 21). Review of bibliography of those eligible records yielded one additional eligible study (20). Finally, three cohort studies (13–15) and six case-control studies (16–21) were eligible for the meta-analysis. Among them, two cohort studies (13, 14) and six case-control studies (16–21) investigated the association between hypothyroidism and risk of PD, while two cohort studies (13, 15) and five case-control studies (16–18, 20, 21) investigated the association between hyperthyroidism and risk of PD. **Figure 1** demonstrates the search methodology and selection process of this study. The characteristics of the included cohort and case-control studies are presented in **Tables 1**, **2**, respectively.

**Table 1:** Main characteristics of the cohort studies included in the meta-analysis.

	Li et al. (13)	Chen et al. (14)
**Country**	Sweden	Taiwan
**Study design**	Retrospective cohort	Retrospective cohort
**Year of publication**	2011	2020
**Main finding(s)**	Hypothyroidism is associated with an increased risk of PD. Hyperthyroidism is associated with an increased risk of PD.	Hypothyroidism is associated with an increased risk of PD.
**Total number of participants**	Total: 310,552 Patients with hyperthyroidism: 34,735 Patients without hyperthyroidism: 275,817 Patients with hypothyroidism: 8,703 Patients without hypothyroidism: 301,849	Patients with hypothyroidism: 4,725 Comparators: 4,725
**Recruitment of participants**	Patients with hypo/hyperthyroidism were identified from the MigMed database located at the Center for Primary Health Care Research, Lund University from 1964 to 2007 Comparators without hypo/hyperthyroidism were identified from the same database.	Patients diagnosed with hypothyroidism between 2000 and 2012 were identified from the 2000 Longitudinal Health Insurance Database which contained claim data and registration files of both ambulatory and inpatient care of one million individuals randomly sampled from the Taiwan National Health Insurance Research Database between 1995 and 2013. Comparators without hypothyroidism were identified from the same database. Comparators were matched to cases by age, sex, index year, and CCI score.
**Diagnosis of hypo/hyperthyroidism**	Hyperthyroidism: Presence of diagnostic codes for Hashimoto/hypothyroidism (ICD-7: 253; ICD-8: 245.1, 243, 244; ICD-9: 245C, 243, 244; ICD-10: E00-03, E06.3) Hyperthyroidism: Presence of diagnostic codes for Graves/hyperthyroidism (ICD-7: 252; ICD-8: 242; ICD-9: 242; ICD-10: E05) in the database	Presence of diagnostic codes for hypothyroidism in the database (ICD-9-CM: 243, 244)
**Diagnosis of PD**	Presence of diagnostic codes for PD in the database (ICD-7: 350; ICD-8: 342.0; ICD-9: 332; ICD-10: G20-21)	Presence of diagnostic code for PD in the database (ICD-9-CM: 332)
**Follow-up period**	Until the development of PD, death, emigration or closing date (December 31^st^, 2007)	Until the development of PD, removal from the National Health Insurance Program of Taiwan, death or the end of 2013
**Average duration of follow-up (years)**	Patients with hypo/hyperthyroidism: N/A Comparators: N/A	Patients with hypothyroidism: 7.1 Comparators: 7.1
**Average age of participants at index date (years)**	Patients with hypo/hyperthyroidism: N/A Comparators: N/A	Patients with hypothyroidism: N/A Comparators: N/A
**Percentage of female**	Patients with hypo/hyperthyroidism: N/A Comparators: N/A	Patients with hypothyroidism: 81.5 Comparators: 81.5
**Variables adjusted in multivariate analysis**	Age, study period, socioeconomic status, region of residence, hospitalization of COPD and hospitalization of alcoholism/alcohol-related liver disease	Age, sex, CCI score, comorbidities and duration of levothyroxine use
**Comorbidities**	N/A	Patients with hypothyroidism: CCI score 0: 75.7%; CCI 1-2: 18.3%; CCI ≥3: 6.0%; brain injury: 4.6%; CVD: 7.9%; HT: 10.5%; dyslipidemia: 19.8%; DM: 12.7% Comparators: CCI score 0: 75.7%; CCI 1-2: 18.3%; CCI ≥3: 6.0%; brain injury: 3.5%; CVD: 6.7%; HT: 10.6%; dyslipidemia: 12.2%; DM: 9.8%
**Newcastle-Ottawa score**	Selection: 4 Comparability: 2 Outcome: 3	Selection: 4 Comparability: 2 Outcome: 3
	**Lin et al. (** 15 **)**	
**Country**	Taiwan	
**Study design**	Retrospective cohort	
**Year of publication**	2021	
**Main finding(s)**	Hyperthyroidism is associated with an increased risk of PD.	
**Total number of participants**	Patients with hyperthyroidism: 8,788 Patients without hyperthyroidism: 8,788	
**Recruitment of participants**	Patients diagnosed with hyperthyroidism between 2000 and 2012 were identified from the 2000 Longitudinal Health Insurance Database which contained claim data and registration files of both ambulatory and inpatient care of one million individuals randomly sampled from the Taiwan National Health Insurance Research Database between 1995 and 2013. Comparators without hyperthyroidism were identified from the same database. Comparators were matched to cases by age, sex, index year, and CCI score.	
**Diagnosis of hypo/hyperthyroidism**	Presence of diagnostic codes for hypothyroidism in the database (ICD-9-CM: 242)	
**Diagnosis of PD**	Presence of diagnostic code for PD in the database (ICD-9-CM: 332)	
**Follow-up period**	Until the development of PD, removal from the National Health Insurance Program of Taiwan, death or the end of 2013	
**Average duration of follow-up (years)**	Patients with hypothyroidism: 8.0 Comparators: 8.0	
**Average age of participants at index date (years)**	Patients with hypothyroidism: N/A Comparators: N/A	
**Percentage of female**	Patients with hypothyroidism: 76.8 Comparators: 76.8	
**Variables adjusted in multivariate analysis**	Age, sex, CCI score, comorbidities and antithyroid therapy	
**Comorbidities**	Patients with hyperthyroidism: CCI score 0: 91.9%; CCI 1-2: 6.7%; CCI ≥3: 1.4%; brain injury: 3.8%; CVD: 4.6%; HT: 13.8%; dyslipidemia: 15.5%; DM: 13.1% Comparators: CCI score 0: 91.9%; CCI 1-2: 6.7%; CCI ≥3: 1.4%; brain injury: 3.7%; CVD: 4.1%; HT: 11.0%; dyslipidemia: 10.2%; DM: 7.8%	
**Newcastle-Ottawa score**	Selection: 4 Comparability: 2 Outcome: 3	

CCI, Charlson Comorbidity Index; CVD, Cerebrovascular disease; DM, Diabetes mellitus; HT, Hypertension; ICD-7, The International Classification of Disease, 7^th^ Revision; ICD-8, The International Classification of Disease, 8^th^ Revision; ICD-9, The International Classification of Disease, 9^th^ Revision; ICD-9-CM, The International Classification of Disease, 9^th^ Revision, Clinical Modification; ICD-10, The International Classification of Disease, 10^th^ Revision; N/A, Not available; PD, Parkinson’s disease.

**Table 2:** Main characteristics of the case-control studies included in the meta-analysis.

	Berger et al. (16)	Bonuccelli et al. (17)
**Country**	United States	Italy
**Year of publication**	1985	1999
**Main finding(s)**	No significant association between hyperthyroidism and PD. No significant association between hypothyroidism and PD.	No significant association between hyperthyroidism and PD. No significant association between hypothyroidism and PD.
**Total number of participants**	Cases with PD: 46 Controls without PD: 46	Cases with PD: 101 Controls without PD: 70
**Recruitment of participants**	Cases: Cases were patients with PD. Controls: Controls without PD were patients with other neurologic disease. Controls were matched to case by age and sex.	Cases: Cases were patients with PD recruited from the Department of Neuroscience, University of Pisa, Italy, from January 1996 to December 1996. Controls: Controls without PD were ambulatory patients with previous stroke recruited from the same institution during the same period. Controls were matched to cases by age and sex.
**Diagnosis of hypo/hyperthyroidism**	N/A	Based of thyroid function tests
**Diagnosis of PD**	N/A	Based on physician diagnosis in the medical record
**Average age of participants (years)**	N/A	Cases: 62.3 Controls: 61.6
**Percentage of female**	N/A	Cases: 47.5 Controls: 52.9
**Variables adjusted in multivariate analysis**	None	None
**Newcastle-Ottawa score**	Selection: 1 Comparability: 1 Exposure: 2	Selection: 2 Comparability: 1 Exposure: 3
	**Tandeter et al. (** 18 **)**	**Munhoz et al. (** 19 **)**
**Country**	Israel	Brazil
**Year of publication**	2001	2004
**Main finding(s)**	No significant association between hyperthyroidism and PD. No significant association between hypothyroidism and PD.	No significant association between hypothyroidism and PD.
**Total number of participants**	Cases with PD: 92 Controls without PD: 225	Cases with PD: 95 Controls without PD: 102
**Recruitment of participants**	Cases: Cases were patients with PD admitted to the geriatric ward of the Soroka University Medical Center, Israel, between 1995 and 1996. Controls: Controls without PD were randomly selected from the same chart review.	Cases: Cases were patients with PD recruited from the Movement Disorders Unit, Neurology Service, Hospital de Clinicas of the Federal University of Parana, Curitiba, Brazil, from August 1997 to August 1998. Controls: Controls without PD were patients followed by other neurological units of the same institution. Controls were matched to cases by age.
**Diagnosis of hypo/hyperthyroidism**	Based on thyroid function tests	Based on thyroid function tests followed by formal assessment by endocrinologists
**Diagnosis of PD**	Based on physician diagnosis in the medical record	Based on the presence of two or more cardinal signs of parkinsonism in the absence of atypical findings pointing to alternative diagnoses in agreement with the UK Brain Bank criteria
**Average age of participants (years)**	Cases: 78.8 Controls: 78.3	Cases: 63.9 Controls: 64.4
**Percentage of female**	Cases: 38.0 Controls: 65.8	Cases: 40.0 Controls: 66.7
**Variables adjusted in multivariate analysis**	None	None
**Newcastle-Ottawa score**	Selection: 2 Comparability: 0 Exposure: 3	Selection: 3 Comparability: 1 Exposure: 3
	**Rugjberg et al. (** 20 **)**	**Kim et al. (** 21 **)**
**Country**	Denmark	Korea
**Year of publication**	2009	2020
**Main finding(s)**	No significant association between hyperthyroidism and PD. No significant association between hypothyroidism and PD.	Hypothyroidism is associated with an increased risk of PD. Hyperthyroidism is associated with an increased risk of PD.
**Total number of participants**	Cases with PD: 13,695 Controls without PD: 68,445	Cases with PD: 5,586 Controls without PD: 22,344
**Recruitment of participants**	Cases: Cases were patients with PD diagnosed between 1986 and 2006 were identified from the Danish National Hospital Register, which contains information on all persons admitted to nonpsychiatric hospitals since 1977. Controls: Controls without PD were randomly selected from the same database. Controls were 1:5 matched to cases by age and sex.	Cases: Cases were patients with PD identified from Korean National Health Insurance Service-National Sample Cohort from 2002 to 2015. Controls: Controls without PD were randomly selected from the same database. Controls were 1:4 matched to cases by age, sex, income and the region of residence.
**Diagnosis of hypo/hyperthyroidism**	Hyperthyroidism: Presence of diagnostic codes for Graves disease in the database Hypothyroidism: Presence of diagnostic codes for Hashimoto thyroidistis in the database	Hyperthyroidism: Patients with presence of diagnostic codes for hyperthyroidism in the database (ICD-10: E05) who were treated ≥2 times Hypothyroidism: Patients with presence of diagnostic codes for hypothyroidism in the database (ICD-10: E02, E03) who were treated ≥2 times
**Diagnosis of PD**	Presence of diagnostic codes for PD in the database (ICD-8: 342; ICD-10: G20)	Presence of diagnostic codes for PD in the database (ICD-10: G20)
**Average age of participants (years)**	Cases: 73.0 Controls: N/A	N/A
**Percentage of female**	Cases: 45.8 Controls: 45.8	Cases: 53.7 Controls: 53.7
**Variables adjusted in multivariate analysis**	None	Obesity, smoking, alcohol consumption, CCI score, thyroid cancer, levothyroxine treatment, goiter and thyroiditis
**Newcastle-Ottawa score**	Selection: 3 Comparability: 1 Exposure: 2	Selection: 4 Comparability: 2 Exposure: 2

CCI, Charlson Comorbidity Index; ICD-8, The International Classification of Disease, 8^th^ Revision; ICD-10, The International Classification of Disease, 10^th^ Revision; N/A, Not available; PD, Parkinson’s disease; UK, United Kingdom.

### Association Between Hypothyroidism and Risk of Parkinson’s Disease

**Figure 2:**
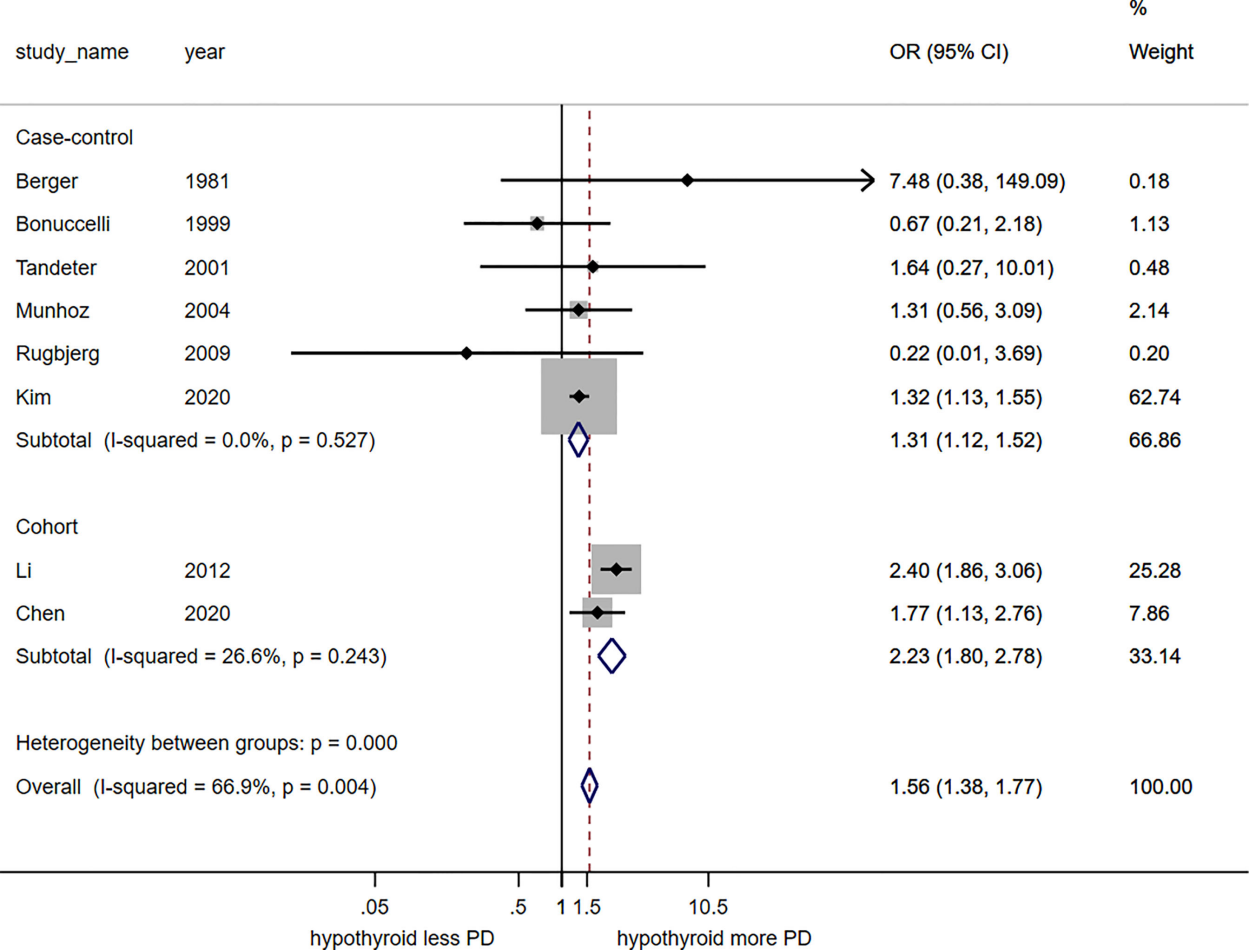
Forest plot of the meta-analysis of risk of Parkinson’s disease in patients with hypothyroidism.

The meta-analysis found a significant association between hypothyroidism and risk of PD with the pooled odds ratio of 1.56 (95%CI, 1.38 – 1.77). This meta-analysis had moderate statistical heterogeneity with I^2^ of 66.9%. Subgroup analysis by study design revealed significant association between hypothyroidism and increased risk of PD among the six case control studies (pooled odds ratio 1.31; 95%CI, 1.12 – 1.52; I^2^ 0.0%) and the two cohort studies (pooled risk ratio 2.23; 95%CI, 1.80 – 2.78; I^2^ 26.6%), as shown in **Figure 2**.

### Association Between Hyperthyroidism and Risk of Parkinson’s Disease

**Figure 3:**
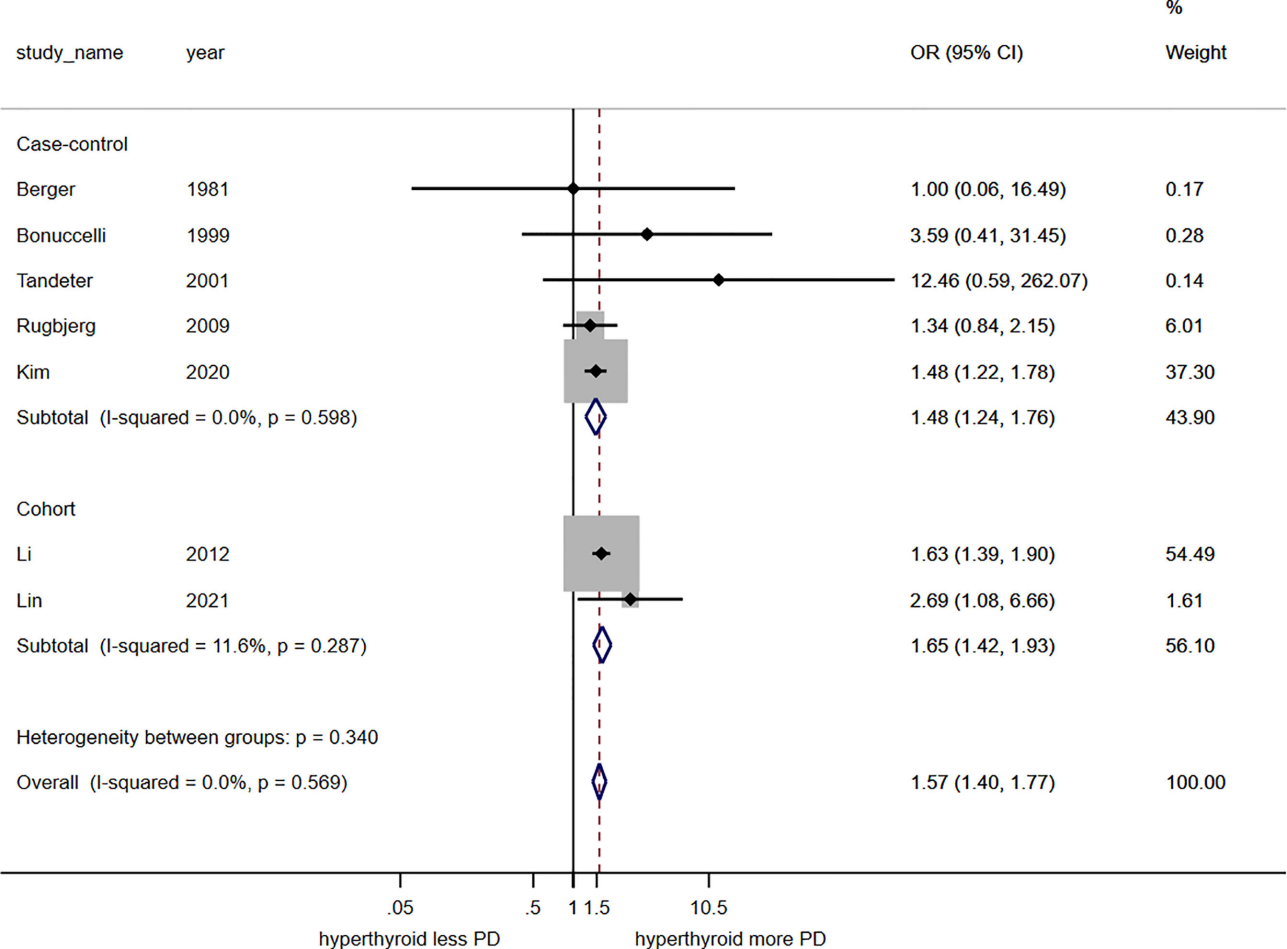
Forest plot of the meta-analysis of risk of Parkinson’s disease in patients with hyperthyroidism.

The meta-analysis found a significant association between hyperthyroidism and risk of PD with the pooled odds ratio of 1.57 (95%CI, 1.40 – 1.77). This meta-analysis had insignificant statistical heterogeneity with I^2^ of 0.0%. Subgroup analysis by study design revealed significant association between hyperthyroidism and increased risk of PD among the five case control studies (pooled odds ratio 1.48; 95%CI, 1.24 – 1.76; I^2^ 0.0%) and the two cohort studies (pooled risk ratio 1.65; 95%CI, 1.42 – 1.93; I^2^ 11.6%), as shown in **Figure 3**.

### Evaluation for Publication Bias

**Figure 4:**
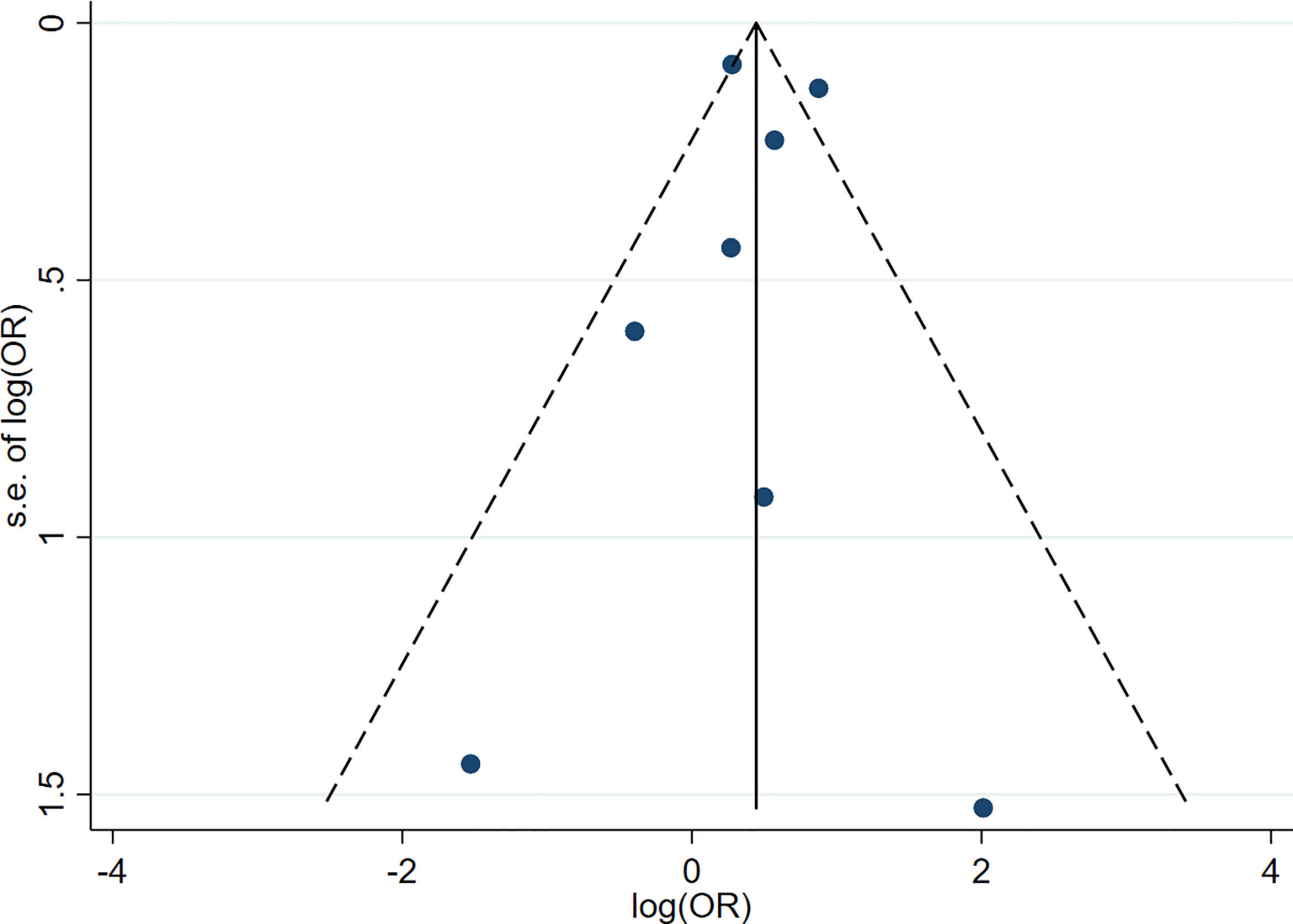
Funnel plot of the meta-analysis of risk of Parkinson’s disease in patients with hypothyroidism.

**Figure 5:**
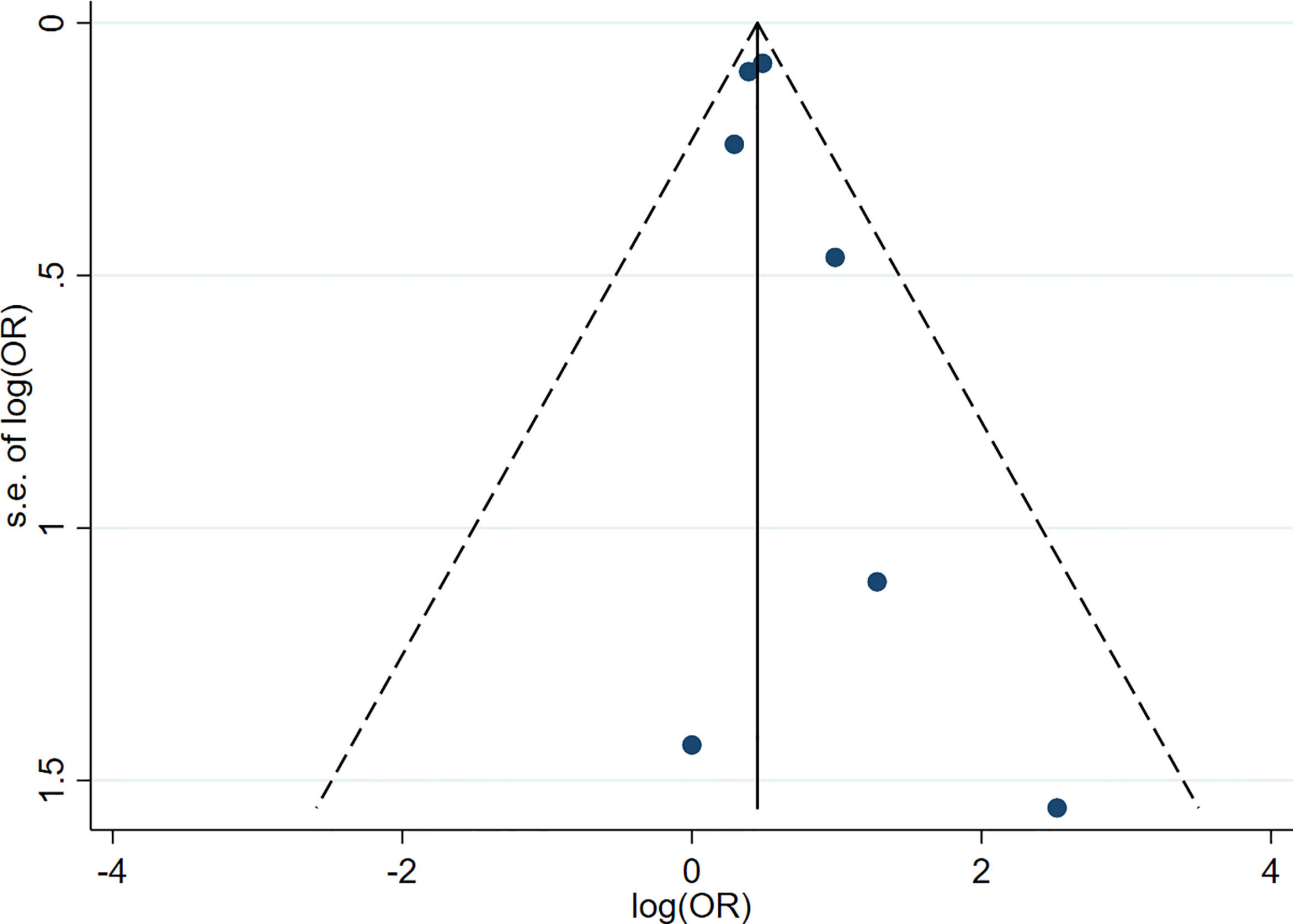
Funnel plot of the meta-analysis of risk of Parkinson’s disease in patients with hyperthyroidism.

Funnel plots were used for assessment for publication bias of the meta-analysis. The funnel plots for both meta-analyses of hypothyroidism and risk of PD (**Figure 4**) and hyperthyroidism and risk of PD (**Figure 5**) were fairly symmetric, which did not indicate presence of publication bias.

## Discussion

This study is the first systematic review and meta-analysis that summarizes all availab e data from cohort and case-control studies that investigated the association between thyroid dysfunction and risk of PD. The pooled analysis revealed that both hypothyroidism and hyperthyroidism were associated with approximately 1.6 times increased risk of PD.

The underlying mechanism of the observed association between thyroid dysfunction and risk of PD is largely undetermined, but there are some possible explanations. First, thyroid hormone is shown to be a key factor for the induction and function of dopaminergic neurons by inducing the expression of the nuclear receptor-related 1 protein (25). This protein is essential for the survival and function of mensencephalic dopaminergic neurons as it appears to modulate the expression of enzymes and transporters that are important for the synthesis and storage of dopamine, including tyrosine hydroxylase, dopamine transporter, vesicular monoamine transporter 2, and l-aromatic amino acid decarboxylase (26). Therefore, it is expected that thyroid hormone deficiency may lead to a decrease in number and function of mesencephalic dopaminergic neuron, which is a pathological characteristic of PD.

On the other hand, thyroid hormone excess can lead to increased cellular metabolism resulting in a high burden of oxidative stress from the mitochondria in multiple tissues including the neuron (27, 28). This process is also known to be the pathophysiological hallmark of PD and therefore is likely to mediate the association between hyperthyroidism and PD (4).

In addition, thyroid dysfunction can result in myopathy, decreased muscle strength and impaired physical performance as thyroid hormone signaling is essential for skeletal muscle development and function (29, 30). Reduced muscle strength is also recognized as one of the neurological presentations in PD (31). Thus, decreased muscle function associated with thyroid dysfunction may lead to overt motor presentation and subsequently diagnosis of PD.

It is also probable that thyroid disease and PD may share a common genetic predisposition since studies have identified variations of several genes including *RASD2*, *WSB1*, *MAPT*, *GIRK2*, *LRRK2* and genes in the NADPH oxidase/dual oxidase family to affect the risk for both thyroid disease and PD (32).

Besides the direct effects of thyroid hormone on dopaminergic neuron and skeletal muscle function and shared genetic risk, it is worth noting that the observed association may be driven by autoimmunity as recent evidence suggests that autoimmune response and neuroinflammation may play a role in the pathogenesis of Parkinson’s disease (13). This supported by the findings from several epidemiologic studies demonstrating an increased risk of PD disorders among patients with autoimmune diseases (13, 33). In addition, studies have suggested that presence of thyroid autoantibodies (anti-thyroid peroxidase and anti-thyroglobulin) are associated with neurodegenerative disorders such as multiple system atrophy and cerebellar degeneration (34, 35). However, whether and how thyroid autoantibodies affect the dopaminergic neurons and risk of PD is unknown.

The results of this study may have some clinical and research implications as they suggest that thyroid hormone signaling plays an essential role in pathogenesis of PD and that overt thyroid dysfunction could be a modifiable risk factor for PD. Further studies should be conducted to determine how duration and degree of severity of thyroid dysfunction affect the risk and severity of PD and to investigate whether treatment to achieve euthyroid status can reverse the risk and severity of PD. It is also worth investigating if subclinical thyroid dysfunction is associated with PD and whether screening for thyroid dysfunction and treatment for subclinical thyroid dysfunction should be recommended in patients with newly diagnosed PD.

This meta-analysis carries some limitations that should be acknowledged. First, in both analyses of hypothyroidism and hyperthyroidism and risk of PD, all except one case-control studies reported no significant association between exposure and outcome. This is likely because most of the case-control studies are of limited quality based on Newcastle-Ottawa score of less than seven (16–18, 20, 36), while most studies that demonstrated significant association are of higher quality (13, 15, 21). Second, many of the included studies relied on diagnosis codes from administrative databases to identify and diagnose thyroid dysfunction and PD (13, 15, 20, 21). Thus, the completeness of case identification and accuracy of the diagnoses of both diseases could be limited. Finally, the relatively small number of included studies in both meta-analyses may jeopardize the validity of the funnel plots.

## Conclusion

This systematic review and meta-analysis found a significant association of both hypothyroidism and hyperthyroidism with an increased risk of PD.

## Data Availability Statement

The original contributions presented in the study are included in the article/**Supplementary Material**. Further inquiries can be directed to the corresponding author.

## Author Contributions 

All authors listed have made a substantial, direct, and intellectual contribution to the work, and approved it for publication.

## Conflict of Interest

The authors declare that the research was conducted in the absence of any commercial or financial relationships that could be construed as a potential conflict of interest.

## Publisher’s Note

All claims expressed in this article are solely those of the authors and do not necessarily represent those of their affiliated organizations, or those of the publisher, the editors and the reviewers. Any product that may be evaluated in this article, or claim that may be made by its manufacturer, is not guaranteed or endorsed by the publisher.
