# Supplementary material for: Thyroid Dysfunction and Risk of Parkinson’s Disease: A Systematic Review and Meta-Analysis
Source: Front Endocrinol (Lausanne). 2022 May 4;13:863281. doi: 10.3389/fendo.2022.863281 (PMC9114488; doi:10.3389/fendo.2022.863281)
Supplement: Supplementary file 1 [file DataSheet_1.docx]

**Supplemental material** – Searching strategy

**Ovid MEDLINE Database**

1. parkinson disease.mp. or exp Parkinson Disease/
2. parkinson's disease.mp.
3. parkinson.mp.
4. idiopathic parkinsonism.mp.
5. primary parkinsonism.mp.
6. hypokinetic rigid syndrome.mp.
7. paralysis agitans.mp.
8. shaking palsy.mp.
9. parkinsonism.mp. or exp Parkinsonian Disorders/
10. movement disorder.mp. or exp Movement Disorders/
11. exp Hypothyroidism/ or hypothyroidism.mp.
12. hashimoto thyroiditis.mp. or exp Hashimoto Disease/
13. hyperthyroidism.mp. or exp Hyperthyroidism/
14. thyrotoxicosis.mp. or exp Thyrotoxicosis/
15. exp Graves Disease/ or graves disease.mp.
16. exp Thyroid Diseases/ or thyroid disorder.mp.
17. thyroid hormone.mp. or exp Thyroid Hormones/
18. thyroxine.mp. or exp Thyroxine/
19. thyroid stimulating hormone.mp.
20. thyrotropin.mp. or exp Thyrotropin/
21. 1 or 2 or 3 or 4 or 5 or 6 or 7 or 8 or 9 or 10
22. 11 or 12 or 13 or 14 or 15 or 16 or 17 or 18 or 19 or 20
23. 21 and 22

**Embase Database**

1. 'parkinson disease'/exp OR 'parkinson disease'
2. 'parkinsonism'/exp OR 'parkinsonism'
3. parkinson
4. 'thyroid'/exp OR thyroid
5. 'hypothyroidism'/exp OR 'hypothyroidism'
6. 'hyperthyroidism'/exp OR 'hyperthyroidism'
7. 'graves disease'/exp OR 'graves disease'
8. 'thyrotoxicosis'/exp OR 'thyrotoxicosis'
9. 'autoimmune thyroiditis'/exp OR 'autoimmune thyroiditis'
10. 'hashimoto disease'/exp OR 'hashimoto disease'
11. hashimoto AND ('thyroiditis'/exp OR thyroiditis)
12. 'thyroxine'/exp OR 'thyroxine'
13. 'levothyroxine'/exp OR 'levothyroxine'
14. 'triiodothyronine'/exp OR triiodothyronine
15. t4
16. 't3'/exp OR t3
17. 'tsh'/exp OR tsh
18. 'thyroid stimulating hormone'/exp OR 'thyroid stimulating hormone'
19. #1 OR #2 OR #3
20. #4 OR #5 OR #6 OR #7 OR #8 OR #9 OR #10 OR #11 OR #12 OR #13 OR #14 OR #15 OR #16 OR #17 OR #18
21. #19 AND #20
